# Inter-Clade Protection Offered by Mw-Adjuvanted Recombinant HA, NP Proteins, and M2e Peptide Combination Vaccine in Mice Correlates with Cellular Immune Response

**DOI:** 10.3389/fimmu.2016.00674

**Published:** 2017-01-09

**Authors:** Nilesh B. Ingle, Rashmi G. Virkar, Vidya A. Arankalle

**Affiliations:** ^1^Hepatitis Division, National Institute of Virology, Pune, India

**Keywords:** H5N1, Mw, adjuvant, vaccine, immunophenotyping, protection, heterologous challenge, M2e

## Abstract

We documented earlier that Mw (heat-killed suspension of *Mycobacterium indicus pranii*) adjuvant when used with conserved antigens, nucleoprotein (NP), and ectodomain of matrix (M2) protein (M2e) provided complete protection against homologous (clade 2.2) virus challenge in mice. The present study extends these observations to inter-clade challenge (clade 2.3.2.1) H5N1 virus and attempts to understand preliminary immunologic basis for the observed protection. Female BALB/c mice immunized with a single or two doses of vaccine formulations (clade 2.2 antigens) were challenged with 100LD50 homologous or heterologous (clade 2.3.2.1) virus. To understand the preliminary immunologic mechanism, we studied proportions of selected immune cell types, immune response gene expression, and Th1/Th2 cytokines induced by antigen-stimulated splenocytes from immunized mice, at different time points. Complete protection was conferred by Mw-HA, Mw-HA + NP, and Mw-HA + NP + M2e against homologous challenge. The protection correlated with IgG2a antibody titers indicating important role of Th1 response. Despite high inter-cladal antigenic differences, complete protection against the heterologous strain was achieved with Mw-HA + NP + M2e. Of note, a single dose with higher antigen concentrations (50 µg HA + 50 μg NP + 50 μg M2e) led to 80% protection against clade 2.3.2.1 strain. The protection conferred by Mw-HNM correlated with induction of IFN-γ, CD8^+^ T cytotoxic cells, and CD4^+^ T helper cells. Mw-adjuvanted HA + NP + M2e combination represents a promising vaccine candidate deserving further evaluation.

## Introduction

Highly pathogenic and rapidly evolving avian influenza virus, H5N1, remains a pandemic threat. Since the first human case of H5N1 infection in Hong Kong in 1997 (1), the number of human cases as of December 2015 increased to 844 across 16 countries with 53% mortality (2). Use of antiviral drugs for the treatment of influenza has limitations because of the emergence of drug-resistant strains of H5N1 virus (3, 4). Evolution of H5N1 virus genetically and antigenically in 10 diversified clades (clade 0–9) (5) underscores the need for a broadly cross-reactive vaccine.

Currently available influenza vaccines mainly rely on HA antigens that do not offer protection against antigenically drifted strains (6). Therefore, the use of the conserved proteins of the virus such as nucleoprotein (NP) and M protein was considered for the generation of broadly reactive immune response and most desired “universal vaccine” (7, 8). Recombinant protein vaccines are regarded as safer alternatives in the pursuit of influenza vaccine development (9). However, lower immunogenicity of these proteins demands the use of potent adjuvants.

Among the protein antigens of influenza, HA is the major target for vaccine development because of the generation of virus neutralizing antibodies (6) that have prophylactic role. Immunization with NP was shown to confer cross protection against influenza A heterosubtypic challenge through CD8^+^ T cell response (10). Non-neutralizing antibodies generated against NP were shown to help viral clearance through antibody-dependent cell cytotoxicity (ADCC) in mice (11). M2e, a 23aa peptide, an ectodomain of M2 protein contains N terminal 9aa epitope (SLLTEVET), which is conserved among 99.3% of all the subtypes of influenza virus (12). To improve the poor immunogenicity of M2e, many researchers have used peptide carrier conjugates (13), multiple antigenic peptides (14), and M2e fusion proteins (15). M2e-specific monoclonal antibodies have been shown to restrict viral growth *in vitro* (16) and also could protect mice after passive transfer (17). The protective role of M2e was shown to involve antibody-mediated inhibition of virus replication (18) and ADCC (19). M2 immunization was shown to provide cross protection against different influenza strains (20).

Following the introduction of H5N1 in India (21), we evaluated HA, NP, and M2e in different combinations using three adjuvants (22). Mw (heat-killed preparation of *Mycobacterium indicus pranii*) was identified for the first time as a promising adjuvant providing complete protection against homologous H5N1 challenge in mice. *M. indicus pranii* (previously known as Mw) (23) was developed as an immunomodulator for the treatment of multibacillary lepromatous leprosy (24) and approved for human use by The Drugs Controller General of India and by US Food and Drug Administration. The immunomodulatory action of Mw mainly comprises of T cell response, activation of Th1 cell subtype, and enhanced secretion of IFN-γ and IL-2 (25). Mw-adjuvanted recombinant human chorionic gonadotropin vaccine elicited enhanced peak antibody titers and duration of antibody response (26).

In this study, we report inter-clade protection offered by Mw-adjuvanted formulation containing HA, NP, and M2e that was predominantly associated with cellular responses.

## Materials and Methods

### Ethics Statement

This study was conducted as per the guidelines and approval of the *Institutional Animal Ethics Committee* (Approval No: IAEC/HEP-14/NIV-54/2012 dated 03/08/2012). H5N1 virus challenge experiments were done at the BSL-3+ biosafety containment facility following the approval of the *Institutional Biosafety Committee* (Approval No: NIVIBSC/27.07.2012/4). The mortality aspects of the protocols were reviewed and approved by the Institutional Animal Ethics Committee. To minimize animal suffering and distress, isoflurane and ketamine–xylazine anesthetics were used during experimental procedures.

### Viruses

H5N1-Navapur-A/chicken/India/33487/2006 (clade 2.2) and JC-2-1 NIVAN 1117307 (clade 2.3.2.1) influenza viruses isolated at National Institute of Virology were used for homologous and heterologous challenge, respectively. The clade 2.2 virus was used for the generation of recombinant proteins as described earlier (22).

### Immunogens

The HA gene was cloned in pFastBac1 and expressed in *Spodoptera frugiperdii* insect cells, and recombinant HA protein was purified using lentil lectin affinity chromatography (GE healthcare, USA). NP gene cloned in pET15b bacterial expression system was used to transform BL21 codon plus (RIL) cells. Recombinant NP protein was purified using Ni^++^ chelated resin (Invitrogen, USA). Synthetic M2e peptide, SLLTEVETPTRNEWECRCSDSSD, was obtained from INBIOS S.r.l, Italy.

### Adjuvants

Mw (5 × 10^9^ cells/ml, heat-killed *M. indicus pranii*) was purchased from Cadila Pharmaceuticals India in the form of “Immuvac.” Mw formulations were prepared by mixing adjuvant and immunogens (1:1 v/v ratio) to make a total of 100-µl dose per mouse containing 50 µl of Mw and 50 µl of immunogens.

### Mice Immunizations

**Figure 1:**
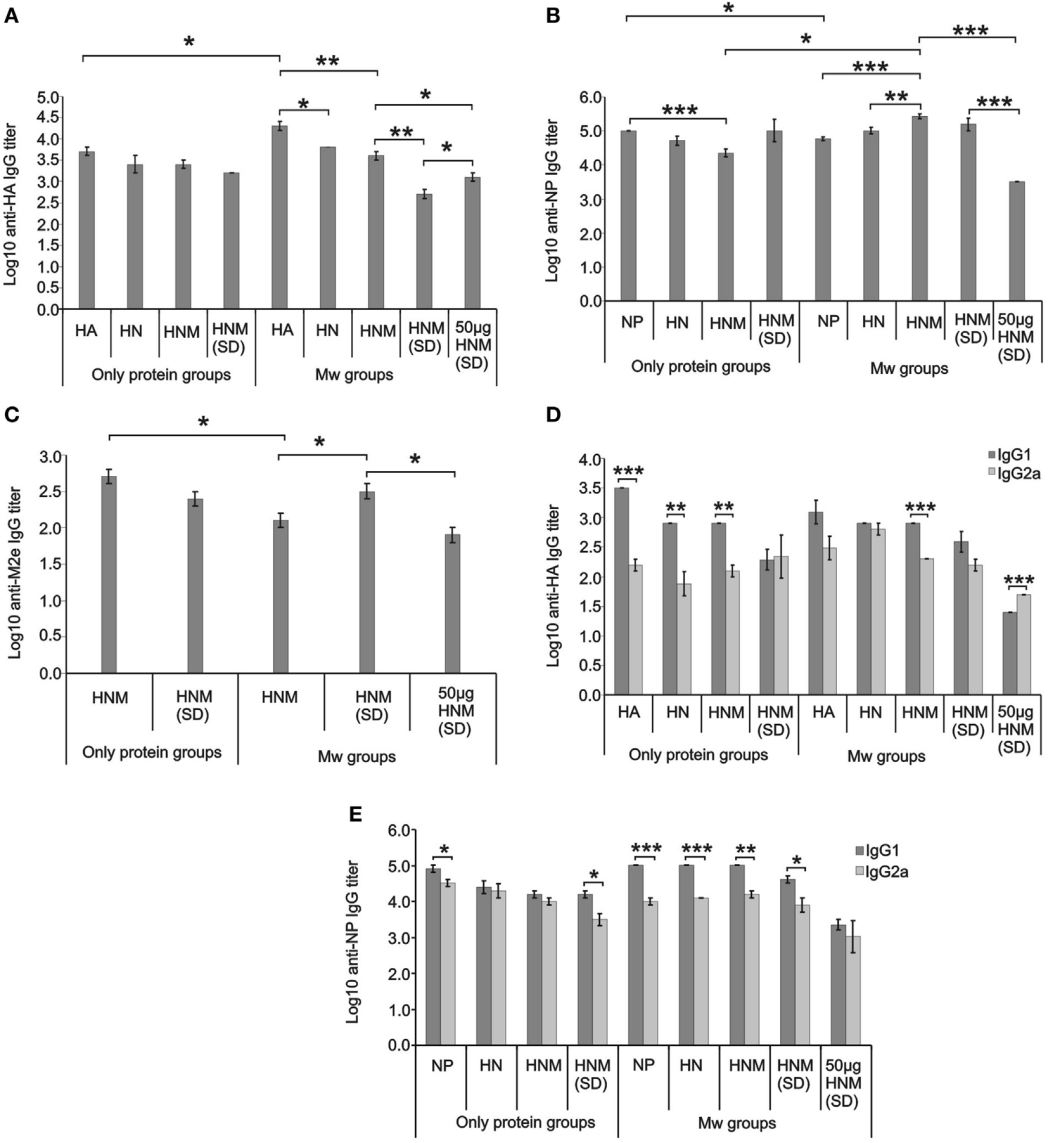
**Antigen-specific serum IgG antibody titers and isotype analysis**. IgG anti-HA **(A)**, anti-NP **(B)**, and anti-M2e **(C)** antibody titers at 3 weeks post-dose-1 (for single-dose immunization) and 10 days post-dose-2 (for two-dose immunization) (prior to virus challenge) in mice immunized with different formulations and IgG1/IgG2a isotype analysis for HA-specific **(D)** and NP-specific **(E)** antibodies at these time points. Abbreviations: HA, hemagglutinin; NP, nucleoprotein; HN, HA + NP; HNM, HA + NP + M2e; HNM (SD), single-dose immunized HA + NP + M2e groups. Data are average of 10 mice per group. Error bars represent SEM. *p < 0.05, **p < 0.01, and ***p < 0.001.

Six to eight week old female inbred BALB/c mice were immunized intramuscularly with 100 µl (50 µl per quadriceps muscle) of single or double doses of respective vaccine formulations given at 3-week intervals (Table 1; Figure 1). Mice were bled before immunization, 3 weeks post immunization, and 10 days post-second dose under isoflurane anesthesia. Control mice received PBS.

**Table 1:** **Hemagglutination inhibition (HI) and IgG-anti-HA, nucleoprotein (NP), M2e titers in mice groups prior to virus challenge**.

Gr. No.	Immunogen	Denotation used in study	Anti-HA IgG antibody titer (GM ± SE)	Anti-NP IgG antibody titer (GM ± SE)	Anti-M2e IgG antibody titer (GM ± SE)	HI titer (GM ± SE)
**Formulations administered as two doses**						
1	10 µg HA + Mw	Mw-HA	20,318 ± 4,267	–	–	64 ± 13
2	10 µg NP + Mw	Mw-NP	–	58,813 ± 10,240	–	–
3	10 µg HA + 10 µg NP + Mw	Mw-HN	6,400 ± 0	102,400 ± 25,083	–	32 ± 6.7
4	10 µg HA + 10 μg NP + 50 μg M2e + Mw	Mw-HNM	4,031 ± 1,067	270,235 ± 50,165	126 ± 33	4.3 ± 6
5	10 µg HA	OP-HA	5,080 ± 1,067	–	–	20 ± 0
6	10 µg NP	OP-NP	–	102,400 ± 0	–	–
7	10 µg HA + 10 μg NP	OP-HN	2,540 ± 1,600	51,200 ± 17,363	–	25 ± 6.7
8	10 µg HA + 10 μg NP + 50 μg M2e	OP-HNM	2,540 ± 533	22,286 ± 7,011	504 ± 133	4.3 ± 6
**Formulations administered as a single dose**						
9	10 µg HA + 10 µg NP + 50 µg M2e + Mw	Mw-HNM	504 ± 133	155,209 ± 79,977	317 ± 67	–
10	10 µg HA + 10 μg NP + 50 μg M2e	OP-HNM	1,600 ± 0	102,400 ± 155,402	252 ± 67	–
11	50 µg HA + 50 μg NP + 50 μg M2e + Mw	50-Mw-HNM	1,270 ± 267	3,200 ± 0	79 ± 17	–

For immunophenotyping experiment, the immunized mice were sacrificed at 24 h/3 weeks post-dose-1 and 24 h/10 days post-dose-2. Harvested spleens were used for immunophenotyping by surface staining, cytokine estimation by cytometric bead array (CBA), and gene expression profiling by Taqman low density array (TLDA). Spleens from unimmunized mice were used for control.

### Serologic Assays

For the detection and titration of IgG-anti-HA, NP, and M2e antibodies, recombinant protein-based ELISA was used as described earlier (22, 27). IgG-subtype analysis was done as described previously (28).

### Hemagglutination Inhibition (HI) Assay

The HI titers were measured on 10 days post-dose-2 as per the guidelines described in WHO manual (29) using receptor destroying enzyme (Denka Seiken UK Ltd., UK), 0.5% turkey erythrocytes, inactivated H5N1-Navapur-A/chicken/India/33487/2006 virus (homologous HI titers), and JC-2-1 NIVAN 1117307 virus (heterologous HI titers).

### Immunophenotyping of Splenocytes

Splenocytes were prepared using standard methods. One million cells were used for antibody labeling. Anti-mouse fluorochrome-labeled antibodies used for different cell types were T cells: anti-CD3-FITC (Clone 17A2), anti-CD4-APC/PE (Clone RM4-5), anti-CD8-APC (Clone 53-6.7); B cells: anti-CD19-APC-Cy7 (Clone ID3), anti-CD45-FITC (Clone RA3-6B2); macrophages: anti-F4/80-FITC (Clone BM8), anti-CD105-APC (Clone MJ7/18); granulocytes: anti-Ly6G-FITC (Clone 1A8), anti-JAML-PE (Clone 4E10); natural killer cells: anti-CD11b-FITC (Clone M1/70), anti-CD49b-APC (HMα2), anti-NKG2D-PE (CX5); and dendritic cells: anti-CD11c-APC (Clone HL3), anti-CD33D1-PE (Clone 33D1). Costimulatory and activation markers used were anti-CD69-PE/FITC (Clone H1.2F3), anti-MHCII-eFluor (Clone M5/114.15.2), anti-CD80-PE (Clone 16-10A1), anti-CD86-FITC (Clone GL1), anti-CD134-PE (Clone OX-86), and anti-CD137L-PE (Clone TKS-1). These anti-mouse fluorochrome-labeled antibodies were purchased from BD Pharmingen, USA/e-Bioscience, USA/Biolegend CA. A minimum of 10,000 events were acquired for every sample after gating splenocyte clusters. Acquisition of samples was carried out on BD FACS ARIA-II flow cytometer, and data were analyzed using BD FACS DIVA software (BD Biosciences, USA).

### *In Vitro* Stimulation of Splenocytes

Splenocytes (one million) from mice groups at 10 days post-dose-2 were cultured and stimulated with 1 µg HA + 1 μg NP + 5 μg M2e at 37°C for 4/24/72 h. The culture supernatants were tested for Th1 cytokines (IFN-γ, IL-2, and TNF) and Th2 cytokines (IL-4, IL-6, IL-10, and IL-5) by using CBA kit (BD Biosciences, USA) as described previously (30). As positive control, splenocytes stimulated with 2.5 µg/well concanavalin A (Sigma, USA) and as negative control, unstimulated cells were used. Cells collected after 4 and 24 h of stimulation were used for surface staining and gene expression analysis by TLDA.

### TLDA

Frozen spleen samples were processed for total RNA extraction and gene expression analysis as described previously (31). cDNA prepared from 500 ng RNA was mixed with 2×PCR master mix (Life technologies, USA) and loaded on the Taqman mouse immune array panel card and run on 7900 Real-Time PCR System (Applied Biosystems, USA). cDNAs from the spleens of naive mice and the mock-immunized (PBS) control mice were used as calibrators. 18s gene was used as the endogenous control. Relative quantification (RQ) values obtained were used to calculate fold upregulation or downregulation. RQ values between 0.5 and 2 were considered normal.

### Virus Challenge

Control and immunized mice with single dose or two doses of the respective formulations (*n* = 10/group) were challenged intranasally with 50 µl of 100 LD50 homologous (H5N1-Navapur-A/chicken/India/33487/2006) or heterologous (JC-2-1 NIVAN 1117307) influenza virus under ketamine–xylazine anesthesia. Single-dose immunized mice were challenged post 3 weeks of immunization, while those receiving two doses were challenged on 10 days post-dose-2. Since the aim of this study was to assess the efficacy of different vaccine formulations against the challenge of H5N1 virus, euthanasia was not used till experimental endpoint [post infection day 14 (PID-14)] to find out if reversal of sickness occurs resulting into survival. Mice were monitored daily for 14 days for weight loss and mortality.

### Lung Viral Load

Three mice per group were sacrificed 72 h post challenge (PID-3) and harvested lungs stored in RNA later at −80°C until tested. RNA was extracted from the lung homogenates using QIAmp viral RNA mini kit (Qiagen, Valencia, CA, USA) according to the manufacturer’s instructions. Quantitation of viral RNA copy number was done by VLA Taqman wet assay real-time Influenza A/H5/H7 detection kit (Applied Biosystems, USA).

### Statistical Analyses

Statistical analyses were performed using the SPSS 20 software (SPSS Inc., IL, USA). To compare antibody titers and proportions of immune cell types, Student’s *t*-test and ANOVA with Tukey’s *post hoc* test were used, while Pearson correlation coefficients were determined by bivariate and partial correlation analyses. PCC values in the range 0.6–0.8 and >0.8 were considered to depict moderate and high correlation, respectively, at *p* < 0.05. Kaplan–Meier survival analysis was performed in GraphPad Prism software. The differences at *p* value <0.05 were considered significant for all the analyses.

## Results

We first determined antibody titers against each immunogen component of all the formulations studied followed by the protective efficacy against homologous virus challenge.

### Immunogenicity of Vaccine Formulations (ELISA)

Table 1 and Figure 1 depict immunogen-specific IgG antibody titers (ELISA) and isotype analysis conducted prior to virus challenge. After first dose, all the immunized mice showed 100% seroconversion against HA, NP, and M2e.

Highest anti-HA-IgG titers were recorded with Mw-HA formulation (20,318 ± 4,267, Figure 1A), while the addition of other antigens, i.e., NP or NP + M2e led to a significant decrease (*p* < 0.05). No adjuvant effect was observed for the combination formulations. With a single dose at the same (*p* < 0.01) or higher (50 µg, *p* < 0.05) Mw-HNM concentration, the titers were lower than two doses of Mw-HNM and were concentration dependent (*p* < 0.05).

Response to NP was distinctly different (Figure 1B). The addition of Mw to NP lowered anti-NP antibody titers (*p* < 0.05), no change was recorded with Mw-HN formulations while the titers increased with Mw-HNM formulation (*p* < 0.05). Among the only-protein formulations, NP elicited significantly higher titers than HNM (*p* < 0.001). Anti-NP titers did not increase with the number of doses for Mw-HNM or HNM. In fact, higher immunogen concentration (50 µg) led to a significant decrease in the antibody titers (*p* < 0.001). Addition of Mw to HNM led to a significant reduction in anti-M2e IgG titers (*p* < 0.05) (Figure 1C). A significant lowering of anti-M2e titers was associated with increasing the number of doses or antigen concentration (*p* < 0.05).

The Th2 response exhibited by non-adjuvanted HA, HN, and HNM formulations was switched to a balanced Th1/Th2 response in Mw-HA, Mw-HN, and the single dose-Mw-HNM/HNM formulations (Figure 1D). As compared to a Th2 response with Mw-HNM, a single dose with fivefold higher antigen concentration led to a distinct Th1 response. Only NP, Mw-NP, Mw-HN, and Mw-HNM induced Th2 response, while a balanced Th1/Th2 profile was seen with HN, HNM, and Mw-adjuvanted single dose with higher antigen formulations (Figure 1E). Since anti-M2e-IgG titers were low in all the groups, isotype analysis was not done.

### HI Titers

**Figure 2:**
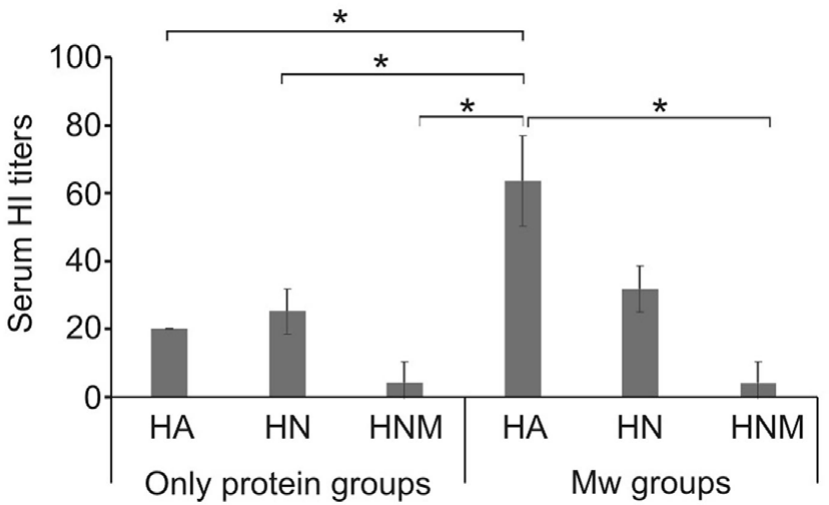
**Serum HI titers (geometric mean + SE) prior to challenge (10 days post-dose-2)**. Abbreviations: HI, hemagglutination inhibition; HA, hemagglutinin; NP, nucleoprotein; HN, HA + NP; HNM, HA + NP + M2e.

Addition of Mw to HA led to significantly higher HI titers (64 ± 13) (Figure 2). Similar to ELISA, addition of NM led to significant decrease in the Mw-HNM group (4.3 ± 6, *p* < 0.05). For HN and HNM formulations, no adjuvant effect was seen. When tested against the clade 2.3.2.1 virus, no HI activity was exhibited by these mice groups.

### Protection of Mice against Homologous and Inter-Clade Virus Challenge and Lung Viral Load

**Figure 3:**
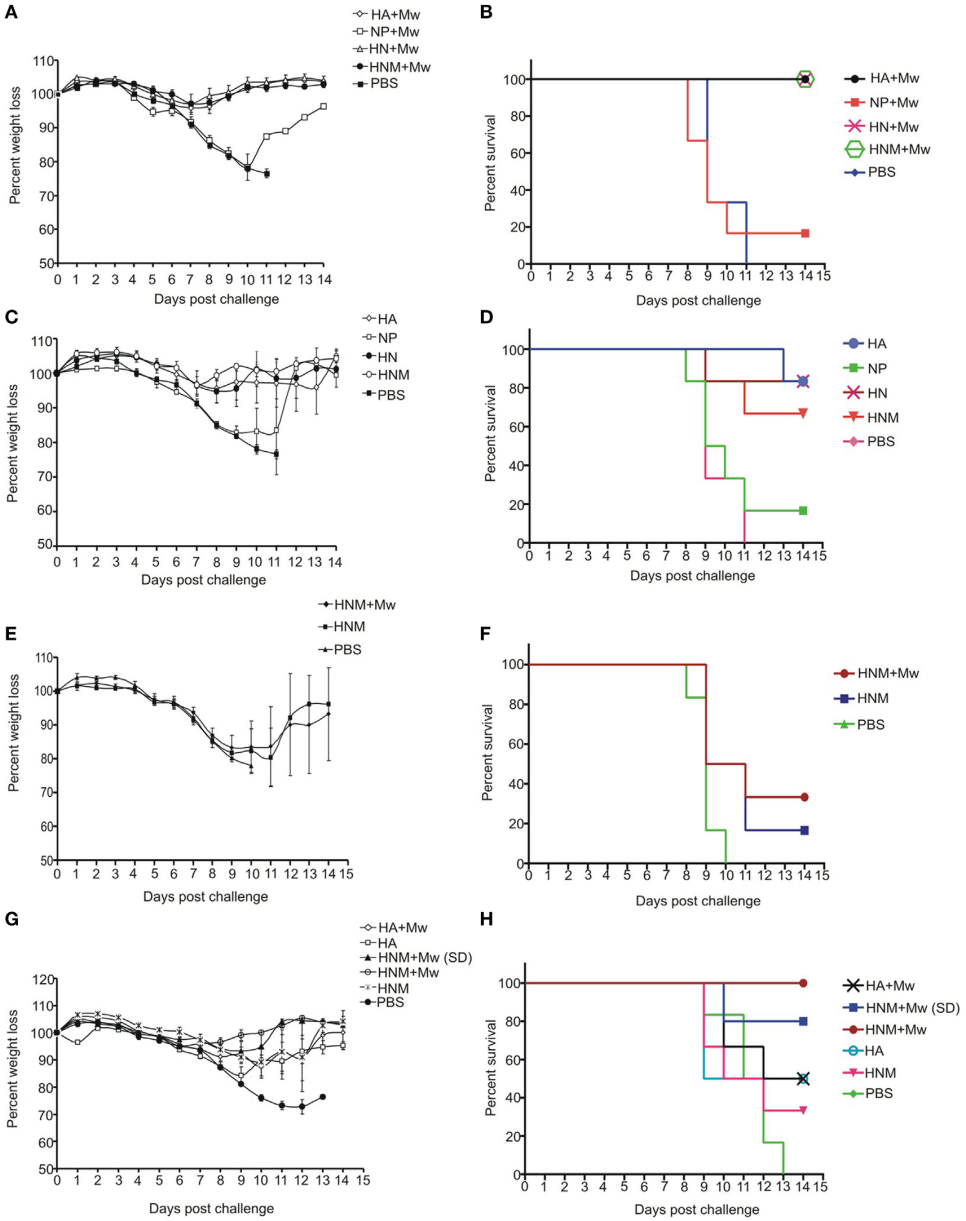
**Morbidity and survival kinetics post challenge**. Immunized and control mice were inoculated intranasally with 100LD50 homologous/heterologous H5N1 virus. The controls received PBS (*n* = 10/group). The groups challenged with homologous virus include Mw formulations **(A,B)**, only-protein formulations **(C,D)**, and single-dose immunized mice groups **(E,F)**. **(G,H)** present mice groups immunized with single- and double-dose receiving challenge with heterologous virus. Abbreviation: HA, hemagglutinin; NP, Nucleoprotein; HN, HA + NP; HNM, HA + NP + M2e; (SD) indicate single-dose immunized groups. % weight loss indicates mean of six mice ± SE.

**Figure 4:**
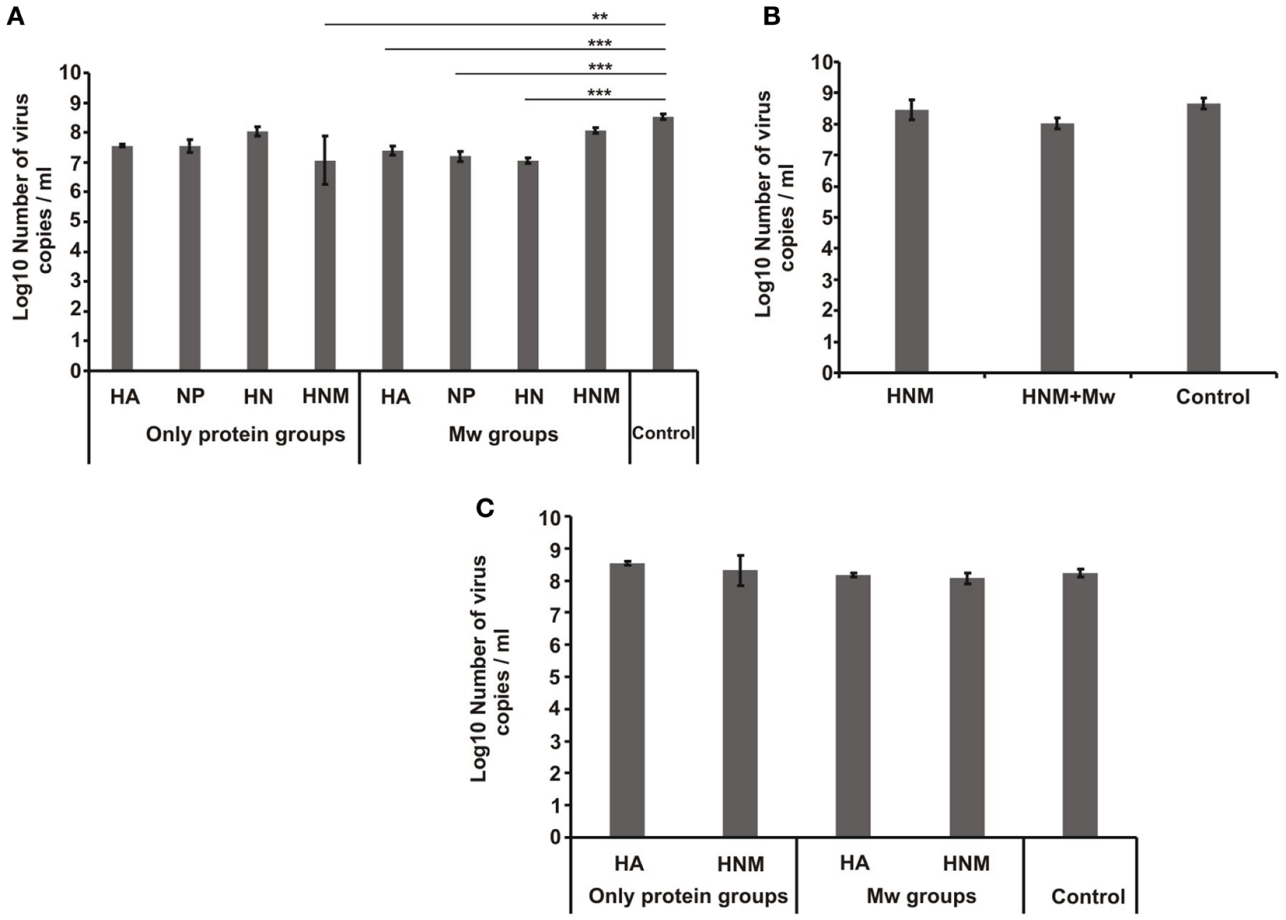
**Lung viral load**. Viral RNA copies per milliliter in the lung suspension of mice at 72 h post infection in **(A)** homologous challenge two-dose immunization, **(B)** homologous challenge single-dose immunization, and **(C)** heterologous challenge groups. Abbreviations: HA, hemagglutinin; NP, nucleoprotein; HN, HA + NP; HNM, HA + NP + M2e. **p < 0.01 and ***p < 0.001.

The mice groups immunized with two doses of different formulations were initially challenged with 100 LD50 homologous viruses. All the three adjuvanted formulations (Mw-HA, Mw-HN, and Mw-HNM) provided complete protection (Figure 3B). The weight loss was minimum in these groups (Figure 3A). Thus, the presence of HA was associated with complete protection. The protection conferred by the respective protein alone formulations was 83, 83, and 67%, respectively (Figure 3D). At 72 h post infection (PID-3), the control mice exhibited 3 × 10^8^ RNA copies/ml lung suspension. Correlation of the reduction in lung viral load and 100% protection was seen with Mw-HA (14-fold, *p* < 0.001) and Mw-HN (29-fold, *p* < 0.001) groups, while protection in Mw-HNM group was associated with no reduction in the viral load (*p* > 0.05, Figure 4A).

Partial protection and considerable weight loss was observed with formulations containing only NP (Figures 3A–D). Interestingly, Mw-NP formulation leading to 21-fold reduction in the lung viral load provided only 17% protection (Figures 4A and 3B). A significant decline in viral load was recorded with HNM (29-fold, *p* < 0.05), while no reduction (*p* > 0.05) was noted with HA, NP, and HN formulations (Figure 4A). Clearly, protection did not correlate with the reduction in lung viral RNA load. With a single-dose HNM immunization, addition of the adjuvant led to higher protection (from 17 to 33%, Figure 3F) with appreciable weight loss (Figure 3E) and comparable lung viral load (Figure 4B).

We selected Mw-HA and Mw-HNM formulations providing 100% protection against the homologous challenge for assessing efficacy against the inter-clade challenge. Irrespective of the adjuvant, HA led to 50% protection (Figure 3H), while 100% protection was achieved by the addition of the conserved antigens; only HNM conferred 33% survival. Importantly, a single dose with higher antigen concentration could provide 80% protection. Weight loss was inversely proportional to the survival rate for all the groups (Table S1 in Supplementary Material, Figure 3G). No reduction in viral load was observed for any of the groups studied (*p* > 0.05) (Figure 4C).

### Correlation of Protection with Parameters Studied

We further attempted to understand the correlation of observed protection and different parameters studied. Bivariate correlation analysis documented highest positive correlation with anti-HA-IgG2a (PCC = 0.918, *p* = 0.001) followed by anti-HA-IgG (PCC = 0.892, *p* = 0.003), anti-HA-IgG1 (PCC = 0.824, *p* = 0.012), and HI titers (PCC = 0.797, *p* = 0.018). Partial correlation analysis identified anti-HA IgG2a (PCC = 0.790, *p* = 0.034), i.e., Th1 response as the only marker correlating with protection. Importantly, HI titers showed high correlation with anti-HA-IgG (PCC = 0.867, *p* = 0.005), anti-HA IgG1 (PCC = 0.827, *p* = 0.011), and anti-HA IgG2a (PCC = 0.840, *p* = 0.009) titers noted in ELISA.

Based on the above results, Mw-HNM was identified as the most efficacious formulation and investigated further.

### Immunophenotyping of Splenocytes from Immunized Mice

As a first step, we determined the proportion of different immune cell types in the spleens of the immunized mice. For this, a few cell types playing crucial role in the generation of innate (macrophages, granulocytes, DCs, and NK cells) and adaptive (T helper, T cytotoxic, and B cells) immune response were studied. Figure S1 in Supplementary Material provides gating strategy.

#### 24  h Post-Dose-1

**Figure 5:**
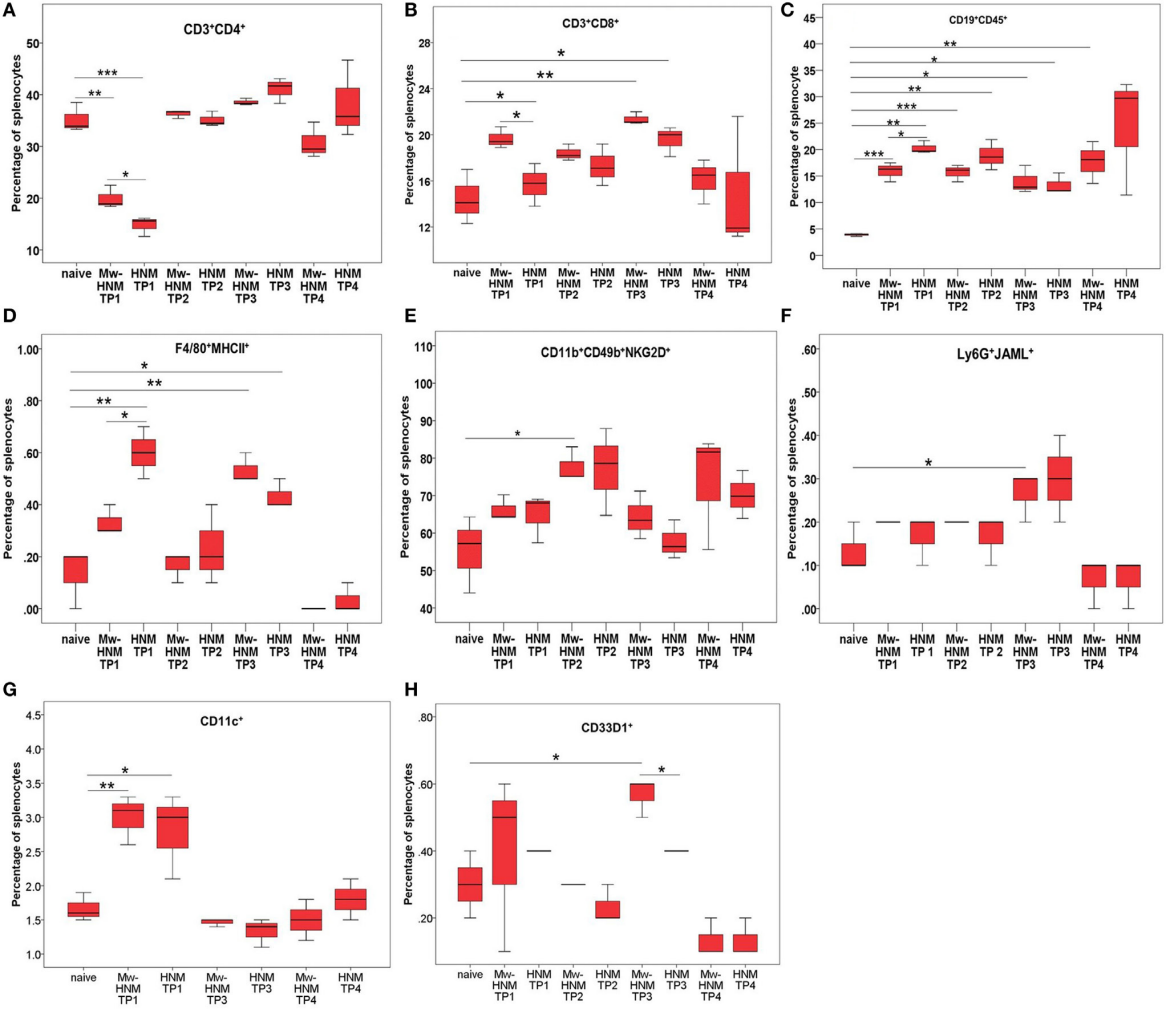
**Enumeration of immune cells by surface staining at different time points post immunization**. Splenocytes from Mw-HMN and HNM-alone immunized mice were used at for immunophenotyping at 24 h post-dose-1 (TP1), 3 weeks post-dose-1 (TP2), 24 h post-dose-2 (TP3), and 10 days post-dose-2 (TP4). **(A)** T helper cells, **(B)** T cytotoxic cells, **(C)** B cells, **(D)** macrophages, **(E)** NK cells, **(F)** granulocytes, and **(G,H)** DCs. Values represent mean % of gated splenocytes ± SD. Abbreviation: HNM, HA + NP + M2e. *p < 0.05, **p < 0.01, and ***p < 0.001.

As compared to the PBS controls, involvement of dendritic cells was seen in both the groups (Figure 5G), while macrophages were restricted to the HNM-alone group (*p* < 0.01, Figure 5D). Both the groups exhibited higher levels of B cells (*p* < 0.05, Figure 5C), while T helper cells reduced (*p* < 0.05, Figure 5A).

Comparison of Mw-HNM and HNM groups showed a significant increase in the levels of macrophages (*p* < 0.05, Figure 5D) in the HNM group, while the proportion of dendritic cells was comparable (Figure 5G). The Mw-HNM formulation was characterized by the higher proportion of both T helper and T cytotoxic cells (*p* < 0.05, Figures 5A,B), whereas lower levels of B cells (*p* < 0.05, Figure 5C) than the HNM category.

#### 3  Weeks Post-Dose-1

When compared to the controls, involvement of NK cells was apparent only in the Mw-HNM group (Figure 5E). In both the groups, proportion of B cells was significantly higher (*p* < 0.05, Figure 5C) than PBS control but was comparable in between.

#### 24  h Post-Dose-2

Following second dose of the respective formulations, the levels of T cytotoxic cells, B cells, and macrophages were higher in both the groups, when compared to the controls (*p* < 0.05, Figures 5B–D), while an increase in granulocytes (*p* < 0.05, Figure 5F) and dendritic cells (CD33D1^+^, *p* < 0.05, Figure 5H) was recorded in Mw-HNM.

When the two formulations were compared, raised levels of dendritic cells in the adjuvanted category (*p* < 0.05, Figure 5H) were recorded.

#### 10  Days Post-Dose-2

At this time point (just prior to challenge), B cells (*p* < 0.01, Figure 5C) and the effector cells for adaptive immunity, i.e., activated T helper (*p* < 0.001), activated T cytotoxic (*p* < 0.001, data not shown) were raised in the Mw-HNM group.

### Immunophenotyping of *In Vitro* Stimulated Splenocytes from the Immunized Mice

#### At 4 H Post-Stimulation

**Figure 6:**
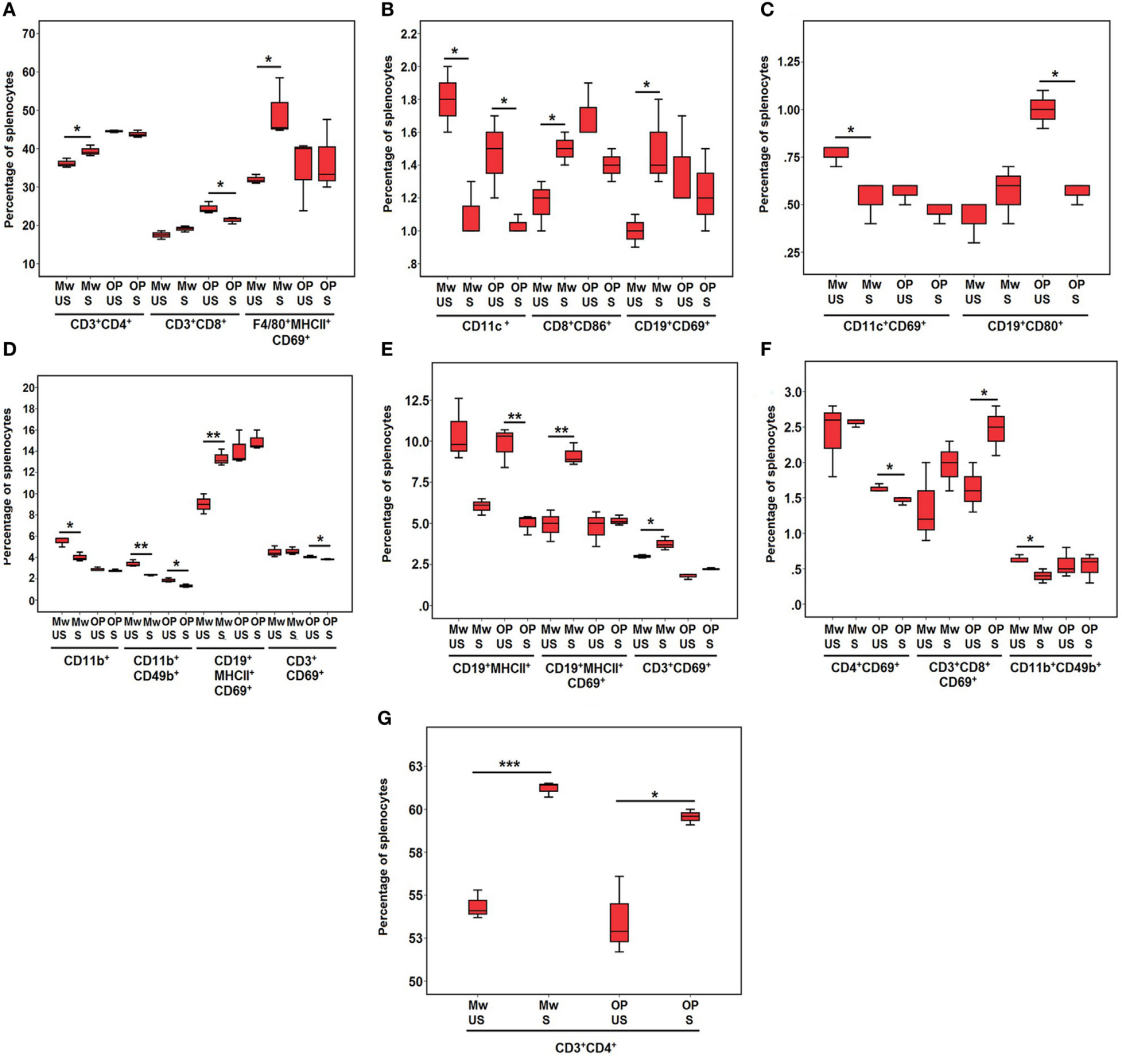
**Enumeration of immune cells by surface staining post antigen stimulation**. Splenocytes from Mw-HMN and HNM-alone immunized mice were stimulated with HNM and were used for immunophenotyping at 4 h post-stimulation **(A–D)** and 24 h post-stimulation **(E–G)**. Abbreviations: US, unstimulated groups; S, stimulated groups; Mw, Mw-HNM groups; OP, only-protein groups (HNM); HNM, HA + NP + M2e. Values represent mean % of gated splenocytes ± SD. *p < 0.05, **p < 0.01, and ***p < 0.001.

T helper cells, T cytotoxic cells with costimulatory molecule (CD8^+^CD86^+^), activated B cells (CD19^+^CD69^+^, CD19^+^MHCII^+^CD69^+^), and activated macrophages (F4/80^+^MHCII^+^CD69^+^) increased in Mw-HNM group (*p* < 0.05) (Figures 6A,D), while the levels of DCs (CD11c^+^, *p* < 0.05), activated DCs (CD11c^+^CD69^+^, *p* < 0.05), and NK cells (CD11b^+^ CD49b^+^, *p* < 0.01) were reduced (Figures 6B–D). No significant increase in any cell type and decreased levels (*p* < 0.05) of activated T cells (CD3^+^CD69^+^), T cytotoxic cells, B cells (CD19^+^CD80^+^), DCs (CD11c^+^), and NK cells (CD11b^+^CD49b^+^) were noted for HNM (Figures 6A–D).

#### At 24 H Post-Stimulation

Continued raised proportion of T helper cells (*p* < 0.001) or activated B cells (CD19^+^MHCII^+^CD69^+^, *p* < 0.01) (Figures 6G,E) and increase in the levels of activated T cells (CD3^+^CD69^+^, *p* < 0.05) was recorded in the Mw-HNM group (Figure 6E), whereas the proportion of NK cells decreased (*p* < 0.05) (Figure 6F). The only-protein group showed a significant rise in the levels of T helper cells (*p* < 0.05) and activated T cytotoxic cells (CD3^+^CD8^+^CD69^+^, *p* < 0.05) (Figures 6G,F), while activated T helper cells (CD4^+^CD69^+^, *p* < 0.05) and B cells (CD19^+^MHCII^+^, *p* < 0.01) decreased (Figures 6F,E).

### Cytokine Estimation and Gene Expression

Culture supernatants of antigen-stimulated splenocytes were used for cytokine estimation. No cytokines were detected at 4 and 24 h post-stimulation in Mw-HNM and HNM groups, while higher levels of IFN-γ (689.5 and 165.8 pg/ml), TNF (260.3 and 210.1 pg/ml), and IL-6 (235.3 and 100.5 pg/ml) were detected at 72 h. A marginal (1.8 pg/ml) and no increase in IL-2 levels was noted in Mw-HNM and only-protein groups, respectively. Mw-induced induction of the signature Th1 cytokine, IFN-γ, was evident. None of the Th2 cytokines tested (IL-4, IL-5) were detected in both Mw-HNM and HNM groups.

**Figure 7:**
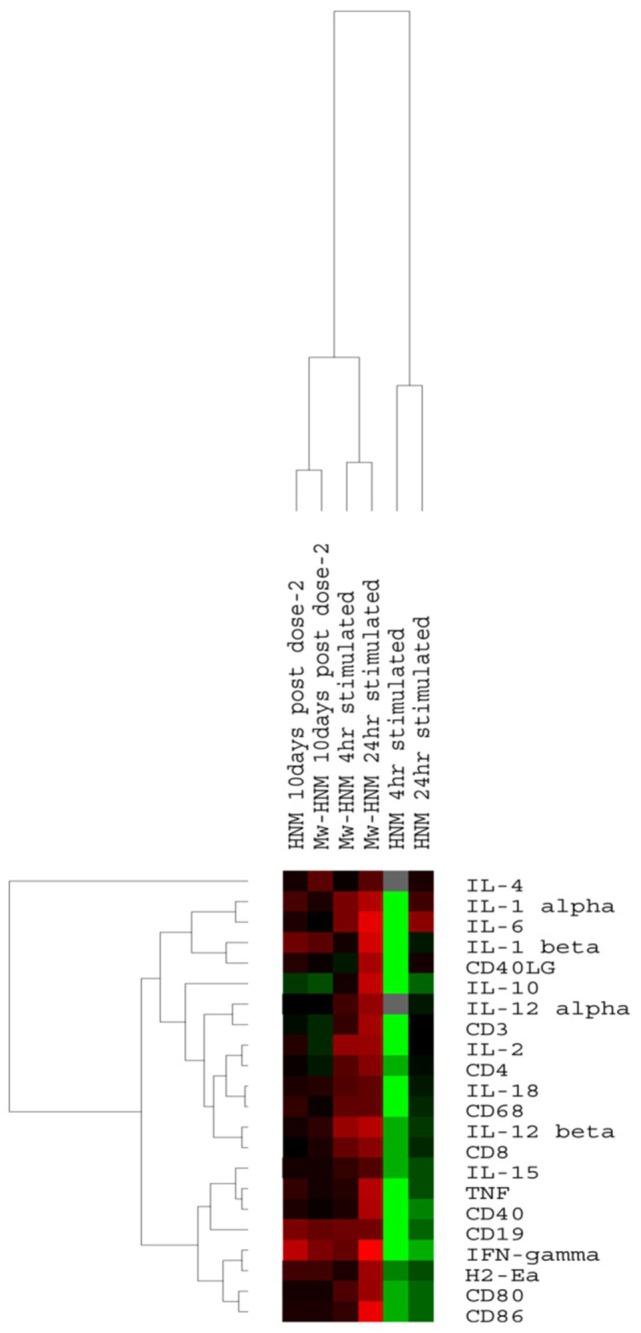
**Heatmap of immune response genes from spleens of immunized mice with Mw-HNM or HNM**. Unstimulated spenocytes collected prior to virus challenge and stimulated with HNM for 4/24 h were studied. The color code: green (downregulation), black (normal), red (upregulation), and gray (unavailability of valid value) of the corresponding gene. Abbreviation: HNM, HA + NP + M2e.

Gene expression analysis was done using splenocytes from the immunized mice (1) prior to virus challenge (unstimulated splenocytes) and (2) *in vitro* cultured and antigen-stimulated splenocytes (4 and 24 h). As evident from the heatmap (Figure 7), stimulated splenocytes from the HNM group formed a distinct cluster, while the unstimulated splenocytes from Mw-HNM and HNM-alone groups and stimulated splenocytes from the Mw-HNM group formed two branches of the second cluster. Table S2 in Supplementary Material presents modulation of mRNA levels of the immune response genes at different time points. Overall, Th1/Th2 and proinflammatory cytokine genes were upregulated in Mw-HNM and downregulated in HNM-alone groups. CD19 was upregulated in the unstimulated Mw-HNM and HNM groups, while the other immune cell surface marker genes were expressed normally. At 4 h post-stimulation, CD19, CD3, CD4, CD8, CD40, and CD80 genes were downregulated in the HNM group. Of these, CD19, CD40, and CD80 genes remained downregulated at 24 h post-stimulation. The mRNA levels of CD19, CD4, CD8, and CD80 were upregulated at both time points in the Mw-HNM group. Of note, FACS (protein) and TLDA (gene) correlated well (Figures 5–7).

## Discussion

Due to ever-changing influenza virus genome and a potential threat of a pandemic with H5N1 virus causing high mortality, there is a definite need for a broad-spectrum vaccine that can be produced rapidly in large quantities and economically viable. HA has been the obligatory immunogen for influenza vaccines, while the two internal proteins, NP and M2, are being evaluated as universal, conserved antigens. Previously, we identified for the first time, utility of Mw adjuvant in providing protection against homologous H5N1 challenge in mice when immunized with several combinations of recombinant HA, NP, and M2e (22). The current study extends this observation to cross-clade challenge and attempts to understand preliminary immunologic mechanism(s) of the adjuvant action. The 5-µg concentration of recombinant antigens was increased to 10 µg and that of M2e peptide to 50 µg per dose.

The protection against 100LD50 homologous challenge varied from 17 to 100% when different formulations were used. The protective role of HA was evident by the minimum weight loss and 100% protection offered by two doses of all the adjuvanted, HA-containing formulations (Mw-HA, Mw-HN, and Mw-HNM). On the contrary, adjuvanted NP provided 17% protection, while M2e alone or in combination with NP was not tried. MVA vectors expressing H5N1-NP alone or coexpressed with H5N1-HA stem protected 50 and 100% mice, respectively, against 42 LD50 challenge of homologous H5N1 virus (32). The difference in the challenge dose may have resulted in the lower NP alone-induced protection noted in our study.

The high immunogenicity of recombinant HA produced in baculovirus system was evident by 83% survival of mice immunized with HA alone. Though the conserved NP and M2e were added to aid this protective immune response, addition of NP did not improve the survival rate (83%), while HA + NP + M2e combination led to a reduction to 67%. It is interesting to note that the addition of NP or NP + M2e to Mw-HA resulted in a gradual decline in both anti-HA-IgG and HI antibodies (Table 1; Figures 1 and 2). Clearly, the protection was not anti-HA antibody-dependent. An increasing trend was seen for anti-NP antibodies, Mw-HNM inducing highest anti-NP titers (*p* < 0.05). M2e elicited uniformly low antibody titers. Taken together, the results suggest non-HA-antibody-associated protection and/or role of NP/M2e in offering protection.

The most promising finding of this study is complete protection of mice immunized with two doses of Mw-HNM (2.2 clade) against intranasal challenge of 100LD50 of the 2.3.2.1 clade strain. Protection with HNM-alone was 33% documenting pronounced adjuvant effect of Mw. We would like to point out here that based on high inter-cladal antigenic differences, Bhat et al. postulated unsuitability of clade 2.2 vaccine in providing protection against clade 2.3.2.1 virus (33). Importantly, in accordance with the documentation of suitability of clade 2.2 vaccine as a good priming vaccine for prime-boost regimen in a pre-pandemic situation (34), Mw-HNM formulation appears worth considering.

Though not strain-matched, both HA and Mw-HA yielded 50% protection that may be attributed to the generation of antibodies against conserved epitopes of globular HA and/or broadly reactive HA-stem epitopes, high affinity and avidity of the antibodies generated, and/or cellular response to HA. Also, 80% protection offered by a single dose of adjuvanted 50 µg immunogen preparation is indeed promising and needs to be explored further.

Though both humoral and cell-mediated immune responses are crucial for protection/recovery in influenza infections, efficacy of the conventional influenza vaccines is judged by HI titers. HI titer of ≥40 is considered protective for the seasonal influenza vaccines. However, lack of universal compliance is recorded (35). HI-independent protection observed by us confirms earlier reports (36–39) and suggests possible role of other immune mechanisms. NP alone with or without Mw provided 17% protection suggesting partial protection in the absence of anti-HA immune response.

A recent study showed that the incorporation of NP of H5N1 virus into HA-NA-M1/M2-VLPs enhanced the immune response and protection against heterologous H5N1 strains that correlated with the generation of high level anti-NP antibodies. In contrast, VLPs without NP provided 50% protection (40). We have used Mw-adjuvanted mixture of HA, NP, and M2e and obtained similar results and are tempted to conclude that Mw-HNM, simpler to prepare, may be an attractive alternative to the VLPs.

Protection against heterotypic strains was earlier shown to be dependent on the preexisting, virus-specific CD4^+^ and CD8^+^ T cells that target the conserved internal proteins of the virus (41, 42). Accordingly, adjuvants enhancing cellular immune responses are of special relevance for H5N1, and Mw is a strong T cell-inducer. We detected antigen-specific cellular responses with unadjuvanted HNM formulation that were adequately enhanced by the adjuvant leading to 100% protection. However, since we did not investigate NP alone, M2e alone, or NP–M2e combination formulations for antigen-specific cellular responses, we are not able to differentiate cellular responses contributed by the respective antigens singly or in combination.

Immunophenotyping of spleens from the immunized mice revealed that high immunogenicity of the Mw-HNM and HNM formulations correlated with an early (24 h post-dose-1) and significantly increased recruitment of DCs, the most efficient antigen-presenting cells, CD8^+^ cells, and B cells. Mw aided the immune response by a further increase in the CD8^+^ T cells and sustained CD4^+^/CD8^+^ T and B cells at the time of virus challenge. Importantly, the HNM-alone-induced lowering of T helper cells that are indispensable for the development of cytotoxic and antibody responses and was compensated by Mw. Protection against lethal challenge with H5N1 was shown to be associated with high CD4^+^ T cell responses against whole-virus antigen but not against recombinant H5 HA, indicating that protection was due to T cell responses against NP but not HA (32). Overall, there is a definite need to establish appropriate correlates of protection for H5N1 virus.

We did not examine specific immune cells for the effect of antigen stimulation. However, restimulation of cultured splenocytes with HNM revealed clear advantage of the adjuvant as evidenced by a rapid increase (4 h post-stimulation) in the frequencies of CD4^+^ T cells, CD8^+^ cells with costimulatory molecule (CD8^+^CD86^+^), activated B cells, and activated macrophages. An inverse relationship between preexisting CD8^+^ T cells, and disease severity was shown during the pandemic of 2009 (43). Association of the robust NP-specific CD8^+^ T cell responses with increased protection against lethal H5N1 challenge indicates possible role of NP (32) and confirms previous findings in humans that CD8^+^ T cell responses against conserved influenza epitopes correlate with protection against influenza (43). M2e alone is a very poor immunogen and needs carrier proteins/adjuvants for the improvement of immunogenicity (44). Several studies have shown superior immune response when M2e was used with other viral antigens and adjuvants (45).

Immune response gene expression analysis showed a concomitant increase in the expression of immune cell surface proteins and higher mRNA levels of CD3, CD4, CD8, CD19, CD40, CD80, CD86 genes in the Mw-HNM and substantial downregulation of these genes in HNM category suggestive of modulation at translational level as well.

Correlation of anti-HA IgG2a with the observed protection emphasizes the usefulness of Mw adjuvant in modulating immune response to Th1 type that is beneficial for protection that was further confirmed by the induction of influenza-specific Th1 cytokines (IFN-γ, TNF, and IL-2) and undetectable levels of Th2 cytokines (IL-4 and IL-10) in the Mw-HNM group. An increase in IFN-γ and IL-2 cytokines and absence of IL-4 in the stimulated T cells (25) and production of high levels of TNF-α (46) have been reported with Mw adjuvant alone in mice. We did observe a correlation of gene and protein expression with Th1 and not Th2 cytokines.

Taken together, our results clearly point out that 2.2 clade-based HNM when adjuvanted with Mw is a promising H5N1 vaccine candidate, and the protection is primarily associated with cellular immune responses. Importantly, Mw is approved by FDA for human use in US and India. A single dose of 50 µg could provide 80% protection qualifying the formulation as a pre-pandemic vaccine. Mw-HNM deserves further experimentations toward a usable pandemic vaccine.

## Author Contributions

NI: planning and performing the experiments/data analysis and preparation of the manuscript. RV: associated with laboratory and BSL-3 experiments. VA: planning and supervision of the study, data analysis, and preparation of manuscript. All authors read and approved the final manuscript.

## Conflict of Interest Statement

The authors declare that the research was conducted in the absence of any commercial or financial relationships that could be construed as a potential conflict of interest.
