# Supplementary figures and images for: Inter-Clade Protection Offered by Mw-Adjuvanted Recombinant HA, NP Proteins, and M2e Peptide Combination Vaccine in Mice Correlates with Cellular Immune Response
Source: Front Immunol. 2017 Jan 9;7:674. doi: 10.3389/fimmu.2016.00674 (PMC5220098; doi:10.3389/fimmu.2016.00674)

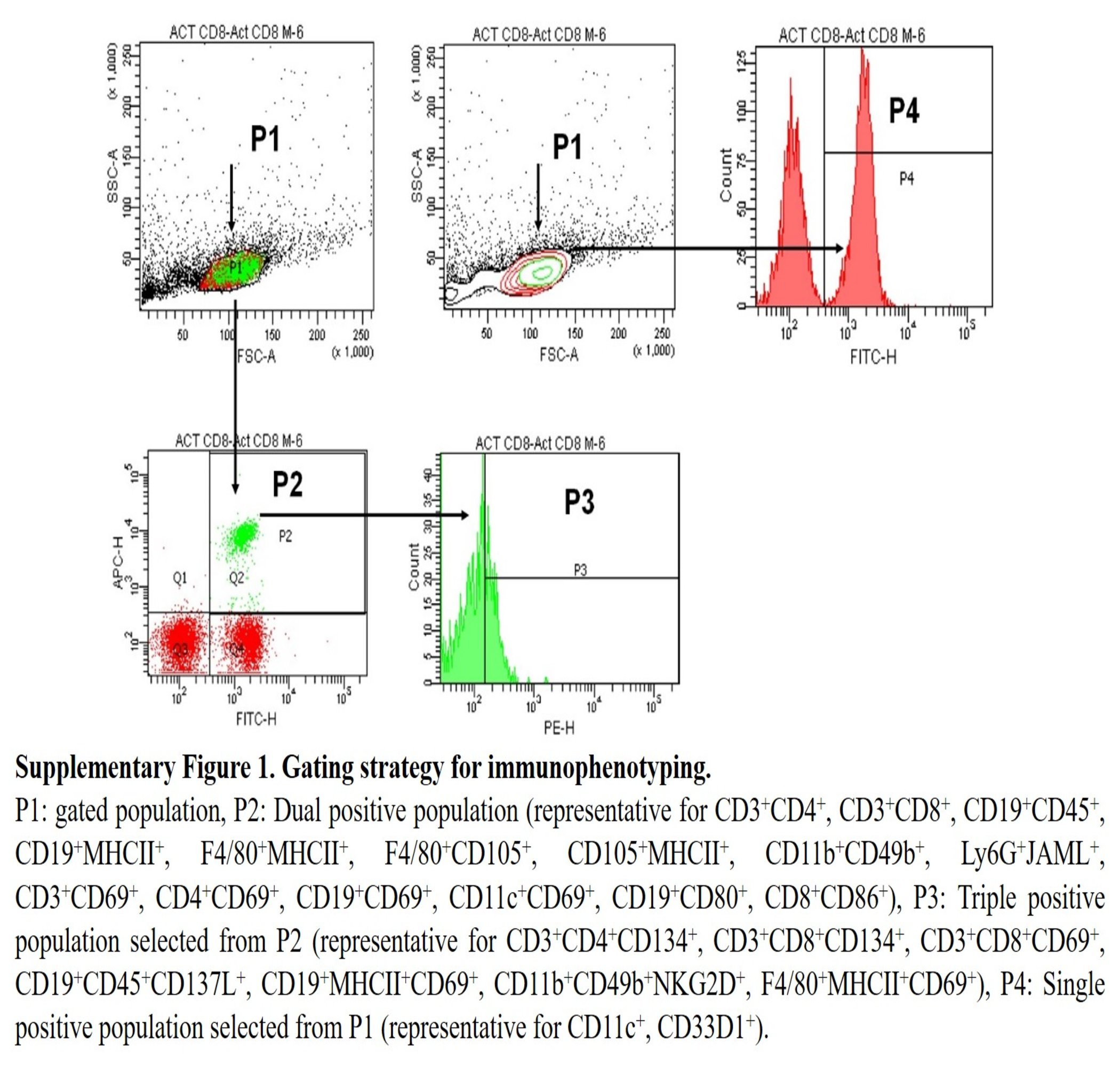

Supplement: Supplementary file 1 [file image_1.jpeg]
