# Supplementary material for: Inter-Clade Protection Offered by Mw-Adjuvanted Recombinant HA, NP Proteins, and M2e Peptide Combination Vaccine in Mice Correlates with Cellular Immune Response
Source: Front Immunol. 2017 Jan 9;7:674. doi: 10.3389/fimmu.2016.00674 (PMC5220098; doi:10.3389/fimmu.2016.00674)
Supplement: Supplementary file 2 [file table_1.docx]

**Supplementary Table 1. Percent survival and weight loss in mice immunized with different formulations and challenged with H5N1 virus**

| **Immunogen** | **Immunization (Dosage)** | **Challenge** | **Percent Survival** | **Average weight loss (%)** |
| --- | --- | --- | --- | --- |
| 10µg HA+ Mw | 2 | Homologous | 100 | 4.0 |
|  | 2 | Heterologous | 50 | 12.5 |
| 10µg HA | 2 | Homologous | 83 | 4.4 |
|  | 2 | Heterologous | 50 | 15.6 |
| 10µg NP+ Mw | 2 | Homologous | 17 | 21.7 |
| 10µg NP | 2 | Homologous | 17 | 17.1 |
| 10µg HA+ 10µg NP+ Mw | 2 | Homologous | 100 | 2.7 |
| 10µg HA+ 10µg NP | 2 | Homologous | 83 | 5.3 |
| 10µg HA+ 10µg NP+ 50µg M2e+ Mw | 1 | Homologous | 33 | 20.1 |
|  | 2 | Homologous | 100 | 2.8 |
|  | 2 | Heterologous | 100 | 4.8 |
| 10µg HA+ 10µg NP+ 50µg M2e | 1 | Homologous | 17 | 19.7 |
|  | 2 | Homologous | 67 | 3.4 |
|  | 2 | Heterologous | 33 | 10.7 |
| 50µg HA+ 50µg NP+ 50µg M2e+ Mw | 1 | Heterologous | 80 | 6.5 |
