# Supplementary material for: Inter-Clade Protection Offered by Mw-Adjuvanted Recombinant HA, NP Proteins, and M2e Peptide Combination Vaccine in Mice Correlates with Cellular Immune Response
Source: Front Immunol. 2017 Jan 9;7:674. doi: 10.3389/fimmu.2016.00674 (PMC5220098; doi:10.3389/fimmu.2016.00674)
Supplement: Supplementary file 3 [file table_2.docx]

**Supplementary Table 2:** **Expression levels of Immune Response Genes in the spleens of immunized mice.**

| **Gene expression (fold changes) at 10 days post-dose 2** | | | | | | |
| --- | --- | --- | --- | --- | --- | --- |
| **Gene** | **Mw-HNM-Immunized** | | | **HNM immunized** | | |
|  | **Unstimulated^*^** | **Stimulated^**^** | | **Unstimulated^*^** | **Stimulated^**^** | |
|  |  | **4hr** | **24hr** |  | **4hr** | **24hr** |
| **Th1 cytokines** |  |  |  |  |  |  |
| IFN-γ | 3.1 ↑ | 2.5 ↑ | 12.7 ↑ | 5.5 ↑ | 13.3 ↓ | 4.2 ↓ |
| IL-2 | 0.7 | 3.9 ↑ | 4.1 ↑ | 1.4 | 13.0 ↓ | 1.0 |
| IL-12 α | 1.0 | 1.8 | 3.9 ↑ | 1.0 | NA | 0.8 |
| IL-12 β | 1.5 | 4.1 ↑ | 5.3 ↑ | 1.2 | 6.6 ↓ | 0.6 |
| IL-15 | 1.2 | 1.6 | 2.1 ↑ | 1.2 | 4.2 ↓ | 2.1 ↓ |
| **Th2 cytokines** |  |  |  |  |  |  |
| IL-4 | 2.3 ↑ | 1.1 | 2.2 ↑ | 1.2 | 21.5 ↓ | 1.3 |
| IL-6 | 1.0 | 3 ↑ | 8.3 ↑ | 1.3 | 10.2 ↓ | 3.6 ↑ |
| IL-10 | 0.5 | 1.2 | 6.0 ↑ | 0.6 | 8.3 ↓ | 2.3 ↓ |
| **Pro-inflammatory cytokines** |  |  |  |  |  |  |
| IL-1 α | 1.3 | 3.0 ↑ | 5.1 ↑ | 1.9 | 7.9 ↓ | 1.8 |
| IL-1 β | 2.3 ↑ | 1.2 | 6.9 ↑ | 2.7 ↑ | 9.2 ↓ | 0.8 |
| IL-18 | 1.4 | 2.1 ↑ | 2.4 ↑ | 1.3 | 7.9 ↓ | 0.8 |
| TNF | 1.2 | 1.4 | 5.2 ↑ | 1.6 | 15.1 ↓ | 0.5 |
| **Immune cell surface antigens** |  |  |  |  |  |  |
| CD3 | 0.7 | 1.6 | 4.5 ↑ | 0.9 | 8.2 ↓ | 1.0 |
| CD4 | 0.8 | 2.2 ↑ | 3.4 ↑ | 1.1 | 6.3 ↓ | 0.9 |
| CD8 | 1.3 | 2.5 ↑ | 3.5 ↑ | 1.0 | 4.5 ↓ | 0.7 |
| CD19 | 2.5 ↑ | 2.7 ↑ | 2.8 ↑ | 3.1 ↑ | 8.8 ↓ | 2.4 ↓ |
| CD40 | 1.1 | 1.3 | 4.9 ↑ | 1.3 | 10.9 ↓ | 3.8 ↓ |
| CD40LG | 1.1 | 0.8 | 4.6 ↑ | 1.4 | 13.8 ↓ | 1.2 |
| H2-Ea | 1.8 | 1.3 | 4.2 ↑ | 1.8 | 3.3 ↓ | 0.5 |
| CD80 | 1.2 | 2.0 ↑ | 4.1 ↑ | 1.2 | 6.4 ↓ | 2.4 ↓ |
| CD86 | 1.3 | 1.6 | 8.5 ↑ | 1.3 | 5.7 ↓ | 2.8 ↓ |
| CD68 | 1.1 | 2.4 ↑ | 2.4 ↑ | 1.6 | 7.6 ↓ | 0.7 |

^*^ Spleen harvested prior to virus challenge and ^**^ Splenocytes harvested prior to virus challenge, cultured and stimulated with HNM for 4hr and 24hr.

RQ values within 0.5-2.0 were considered within normal range.

↑ = Upregulation as compared to control, ↓ = Downregulation as compared to control

NA = values not available.

Abbreviation: HNM indicate HA+NP+M2e formulation.
